# Fired Cartridge Case Identification Using Optical Images and the Congruent Matching Cells (CMC) Method

**DOI:** 10.6028/jres.119.023

**Published:** 2014-11-06

**Authors:** Mingsi Tong, John Song, Wei Chu, Robert M Thompson

**Affiliations:** 1 School of Mechatronics Engineering, Harbin Institute of Technology, Harbin 150001, China; 2 National Institute of Standards and Technology, Gaithersburg, MD 20899, USA; 3 X-wave Innovation, Inc., Gaithersburg, MD 20878, USA

**Keywords:** ballistics identification, cartridge case, Congruent Matching Cells (CMC), correlation cells, image processing

## Abstract

The Congruent Matching Cells (CMC) method for ballistics identification was invented at the National Institute of Standards and Technology (NIST). The CMC method is based on the correlation of pairs of small correlation cells instead of the correlation of entire images. Four identification parameters – *T*_CCF_, *T*_θ_, *T*_x_ and *T*_y_ are proposed for identifying correlated cell pairs originating from the same firearm. The correlation conclusion (matching or non-matching) is determined by whether the number of CMC is ≥ 6. This method has been previously validated using a set of 780 pair-wise 3D topography images. However, most ballistic images stored in current local and national databases are in an optical intensity (grayscale) format. As a result, the reliability of applying the CMC method on optical intensity images is an important issue. In this paper, optical intensity images of breech face impressions captured on the same set of 40 cartridge cases are correlated and analyzed for the validation test of CMC method using optical images. This includes correlations of 63 pairs of matching images and 717 pairs of non-matching images under top ring lighting. Tests of the method do not produce any false identification (false positive) or false exclusion (false negative) results, which support the CMC method and the proposed identification criterion, *C* = 6, for firearm breech face identifications using optical intensity images.

## 1. Background

**Fig. 1:**
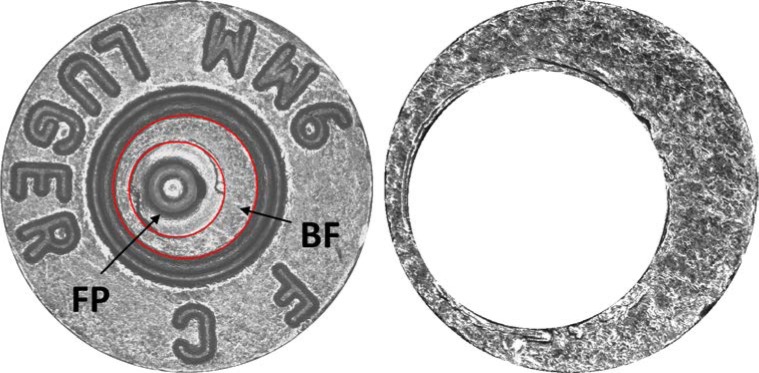
Left one is the image of bottom of cartridge case. Breech face impression (shown as BF) is the area between two red circles. FP indicates impression left by the firing pin. Right one is the image of breech face after trimming.

The toolmarks found on fired bullets and cartridge cases are important physical evidence in shooting incidents. Through comparing microscopic images of toolmarks, forensic examiners can identify sources of firearms, and the results can assist detectives in their investigation. During firing, microscopic individualizing toolmarks are impressed by the breech face of the pistol slide onto the soft metal of the cartridge primer (Fig. 1). Any breech face surface in contact with the primer surface will press a negative impression onto the cartridge case primer, and these toolmarks are the basis for possible identification to a particular pistol slide. Fired cartridge cases collected at crime scenes and test fired firearms are manually compared in ballistic database search systems. In recent years, researchers and forensic scientists have been studying and improving automatic forensic identification systems. For cartridge cases, many ballistic evidence search methods compare whole breech face images to make correlations [1, 2]. But in many cases, not all areas of the breech face impression are related to the breech face topography of gun slide. The detail in these non-related areas may reduce the accuracy of the overall correlation score and rank in a database search.

In 2012, NIST proposed a new method of firearm identification, called the Congruent Matching Cells (CMC) method, which is based on 3D topography measurements on correlation cells with the ultimate goal of providing objective and high-accuracy ballistics identifications and evidence searches [3]. The CMC method was proposed to improve correlation accuracy by automatically identifying the “valid correlation areas” and eliminating the “invalid correlation areas” from consideration. The CMC method uses four identification parameters – *T*_CCF_, *T*_θ_, *T*_x_ and *T*_y_ to determine and qualify the congruent matching cell pairs. A numerical identification criterion *C* = 6 was suggested as a preliminary identification criterion for identifying toolmark impression topographies resulting from being fired from the same firearm [3].

The initial validation tests for the CMC method were performed at NIST by correlating breech face images of 780 pair-wise 3D topography images acquired from 40 cartridge cases fired from handguns using 10 consecutively manufactured pistol slides [4]. Consecutively manufactured slides represent a particular population where the same tools and machining processes are utilized back-to-back on one slide after another. This represents a situation where there would be the most similarity seen between slides [5]. The correlation results strongly support the proposed CMC method and the numerical identification criterion *C* = 6 for ballistics identifications without any false identification (false positive) or false exclusion (false negative) results. Since most images stored in current national databases are in an optical intensity (grayscale) format, there exists a practical requirement to validate the effectiveness of the CMC method when applied to optical intensity images. For this purpose, images of the same set of samples were captured using an optical microscope and then correlated using the proposed CMC method. The basic concepts, experiment procedure, data analysis, and results are described in the following sections.

## 2. Basic Concepts

### 2.1 Correlation Cells and Congruent Matching Cell (CMC) Pairs

The surfaces of the bullets and cartridge cases when fired or ejected from a firearm include both the “valid” and “invalid” correlation areas [3]. A valid correlation area contains “individual characteristics” [6] of the ballistic signature that can be used effectively for identification. An invalid correlation area does not contain individual characteristics of the firearm’s ballistic signature due to incomplete contact with the firearm surface or random prefired surface features on the cartridge case or bullet, and should be excluded from comparison [6]. Assuming that two ballistic images from casing A and casing B originate from the same firearm, both contain valid and invalid correlation areas. When A and B are correlated with each other, their common valid correlation area is the overlap of their individual valid correlation areas, which comprises only part, and sometimes even a small part, of the individual valid correlation areas of A and B. If the correlation is performed on the whole area of A and B, a quantitative measurement of correlation may be relatively low because large invalid correlation areas are included in the correlation. If instead, the correlation area is divided into cells for correlation, the valid correlation cells can be identified and the invalid correlation cells can be excluded from correlations. This procedure can significantly increase the quantitative measure of correlation.

### 2.2 Four Identification Parameters

**Fig. 2:**
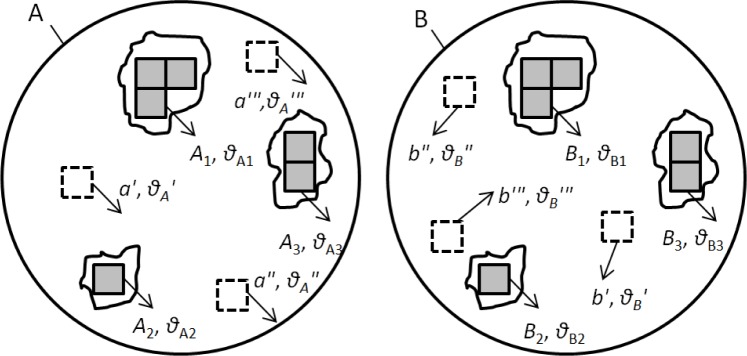
Topographies A and B, originating from the same firearm, are registered at their maximum correlation position. There are three sets of six correlation cell pairs *A*_1_, *A*_2_, *A*_3_ and *B*_1_, *B*_2_, *B*_3_ located in their common valid correlation areas (as shown by six solid cells). The other cell pairs *a*′, *a*″, *a*′″… and *b*′, *b*″, *b*′″… are located in the invalid correlation area (as shown by the dotted cells).

A correlation cell is a sub-area of the surface topography that contains a sufficient quantity of contours, peaks, and valleys so that an assessment of topography similarity and firearm identification can be made. If topographies A and B, originating from the same firearm, are registered at their maximum correlation position (Fig. 2), the registered cell pairs located in their common valid correlation area (as shown by the solid cell pairs *A*_1_, *A*_2_, *A*_3_… and *B*_1_, *B*_2_, *B*_3_…) are characterized by four identification parameters:

*T*_CCF_ – High correlation values quantified by the cross correlation function maximum *CCF*_max_ [7]; *T*_θ_ – The same registration angles *θ* for all correlated cell pairs in topography A and B; and *T*_x_ and *T*_y_ – The same *x*–*y* spatial distribution pattern between the correlated cells array *A*_1_, *A*_2_, *A*_3_… and *B*_1_, *B*_2_, *B*_3_… which is characterized by their “congruent” *x*–*y* spatial distribution pattern on the correlated topographies A and B.

Alternatively, if the registered cell pairs come from the invalid correlation areas of A and B originating from the same firearm (such as the dotted cells *a*′, *a*″, *a*′″… and *b*′, *b*″, *b*′″… in Fig. 2), or if they originated from different firearms, their correlation value *CCF*_max_ would be relatively low, and their cell arrays would show different *x*–*y* distribution patterns and different registration angles *θ* [3].

The CMC method registers the correlation cell using four correlation parameters: *CCF*_max_ (cross correlation function maximum), registration angle *θ*, and translation distance *x* and *y* at their maximum correlation position. It is anticipated that if breech face impressions on two cartridge cases A and B originate from the same firearm, the correlation cell pairs located in their common valid correlation area will show high *CCF*_max_, the same *θ* and the same (*x*, *y*) within their corresponding thresholds. On the other hand, if the cell pairs come from the invalid correlation areas of A and B originating from the same firearm, or if they are from different firearms, their *CCF*_max_ must be relatively low, and the *θ* and (*x*, *y*) of these cell pairs will be significantly different [7].

Inspired by the numerical identification criterion of the Consecutively Matching Striate (CMS) method proposed by Biasotti and Murdock for identification of the bullet striation signatures [8], the numerical identification criterion for the CMC method is proposed as *C* = 6 [3], i.e., when the CMC number of the correlated topographies A and B is equal to or more than *C* = 6, A and B are identified as having been fired from the same firearm.

## 3. Experiment

### 3.1 Test Samples

The validation tests use the same fired cartridge case collection for topography image tests [4] that originated from a study by the Miami Dade Crime Laboratory [5]. Forty cartridge cases fired from pistols using 10 consecutively manufactured slides were correlated with one another. The test set includes 20 known cartridge cases (2 per slide) for training and 20 “unknown” cartridge cases for blind testing. The optical intensity images were captured using a comparison microscope with a top-ring lighting source that provides uniform lighting conditions. After trimming processing to remove unrelated correlations areas, the final image size is reduced to 700 × 700 pixel with an estimated pixel spacing of 5.0 μm. A Gaussian smoothing filter (standard deviation *σ* = 1) was implemented first to remove high frequency noise [9].

### 3.2 Implementation of CMC Method

**Fig. 3:**
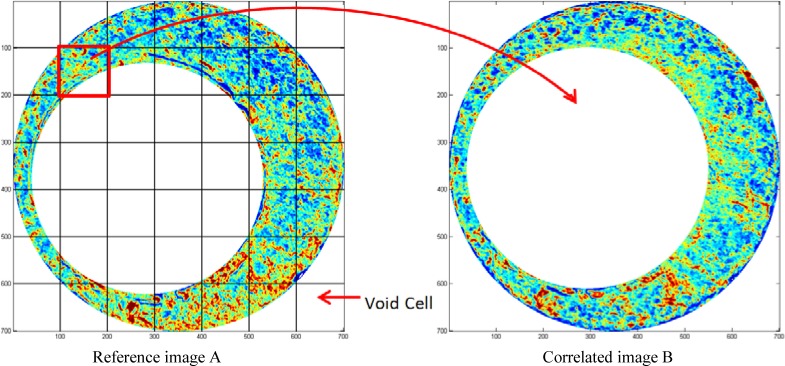
Correlation scheme using the CMC method.

The images for correlation are divided into 7 × 7 cell array. The cell size is set as 100 × 100 pixels or approximately 0.5 mm × 0.5 mm. The actual number of cells involved in the correlation is less than the nominal number of cells, because some void cells that contain no or very few data points are excluded (Fig. 3). For a pair of tested images A and B, each valid cell in the reference image A scans the correlated image B at each rotated position of image B in order to find their position of maximum correlation. In this experiment, the rotation step is 3°. Once the procedure is completed, the cell similarity metric (*CCF*_max_ value) and the image consistency metrics (consisting of the registration angle *θ* and the translation distances in *x*, *y*) are recorded.

### 3.3 A Fast Correlation Method

**Fig. 4:**
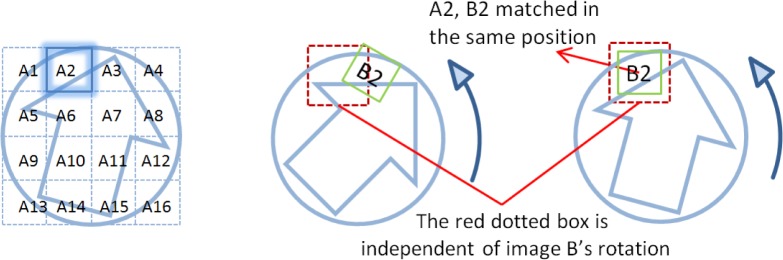
Correlation scheme using the fast CMC method.

Correlations of each cell in the reference image against the whole correlated image are a time consuming process. To reduce the computation time, a fast correlation method is developed. Since both the pistol slide chamber and cartridge case are round-shaped surfaces, when two cartridge cases are fired from the same pistol, their matched cell pairs at the breech face must be located at an equal radial distance from the center axis. Therefore, during the correlation process each correlation cell in image A doesn’t need to be scanned through the whole area of the correlated image B. It is only necessary to scan a local area of the correlated image B within the same radial distance as the reference cell in image A. An example is shown in Fig. 4: A and B is a pair of known matching (KM) images. *A*_2_ and *B*_2_ represent the matched cell pair. During the correlation process, cell *A*_2_ only correlates the red dotted area in each rotated position of image B. With the rotation of image B, cell *A*_2_ can only match *B*_2_ at a specific rotation position where the whole image B matches image A. The size of the actually scanned area on image B, as indicated by the red dotted box, is bigger than the cell size as shown in image A. This can ensure the correct matching cell will not be missed in the correlation. In this experiment, the size of the scanned area in reference image B is 300 × 300 pixel, or 1.5 mm × 1.5 mm approximately. With the fast algorithm applied, the correlation speed can be increased about ten times compared with the time needed scanning the whole area of the correlated image B.

## 4. Experimental Results and Analysis

### 4.1 Thresholds Optimization

**Fig. 5:**
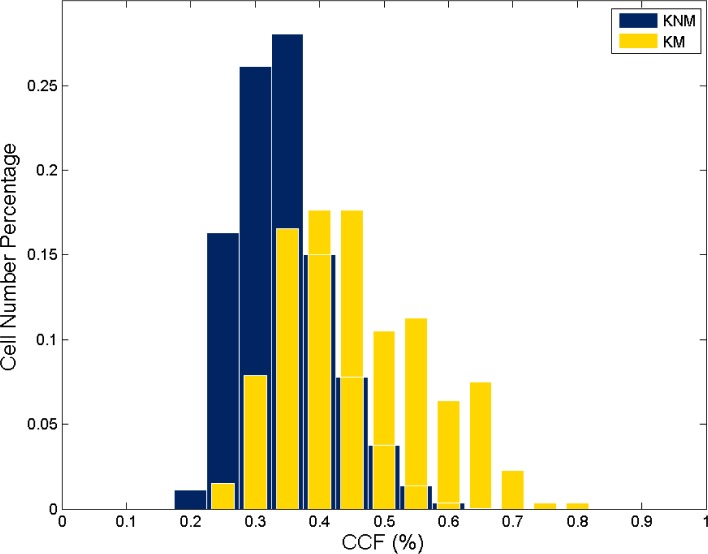
*CCF*_max_ distribution histograms for all cell pairs of 10 KM and 180 KNM correlations.

The 20 training cartridge cases are correlated first, that includes 10 known matching (KM) and 180 known non-matching (KNM) correlations. Excluding those void cell pairs that do not contain sufficient data points, the correlations were implemented for 266 cell pairs of 10 KM correlations and 4794 cell pairs of 180 KNM correlations. Correlation results obtained from the training set are used to determine the thresholds. The *CCF*_max_ distribution histograms for all cell pairs are shown in Fig. 5. It can be seen that there is a large overlap between the distributions of *CCF*_max_ values of KM and KNM cell pairs. In order to include most matching cell pairs for correlation (more than 95 %), a relatively low threshold of *T*_CCF_ = 25 % is selected.

**Fig. 6:**
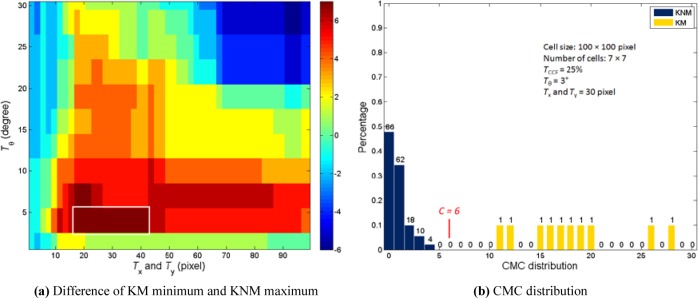
190 correlation results based on *T*_CCF_ = 25 %.

For the optimal determination of the *T*_θ_, *T*_x_ and *T*_y_, a program to sweep all different *T*_θ_, *T*_x_ and *T*_y_, combination at each given *T*_CCF_ value is implemented. By searching the maximum difference between the KNM and KM distributions, or the biggest gap between the maximum CMC number of KNM group and the minimum CMC number of KM group, the optimized thresholds *T*_θ_, *T*_x_ and *T*_y_ can be determined. For the set of 20 training cartridge cases, Fig. 6a shows the CMC differences calculated from the selected 10 KM and 180 KNM correlations with *T*_CCF_ = 25 %. In Fig. 6a, the abscissa represents the thresholds *T*_x_ and *T*_y_ and the ordinate represents the threshold *T*_θ_. The CMC differences (or the gap between the KNM and KM distributions, see Fig. 6b) are scaled by the color bar. Selecting the thresholds in the area of the white frame where the gap achieves the maximum at *T*_θ_ = 3°, *T*_x_ and *T*_y_ = 30 pixel or 0.15 mm can ensure the optimal correlation results.

### 4.2 Experimental Results and Analysis

Figure 6b shows the CMC distribution histograms for both KM and KNM groups under the optimized thresholds. It can be seen that the KM and KNM distributions are well separated. The maximum CMC number of the 180 KNM correlations is 4, and the minimum CMC number for the 10 KM correlations is 11. The gap between them is 6, which indicates that no false identification (false positive) or false exclusion (false negative) identification happens if the numerical identification criterion is set at *C* = 6.

**Fig. 7:**
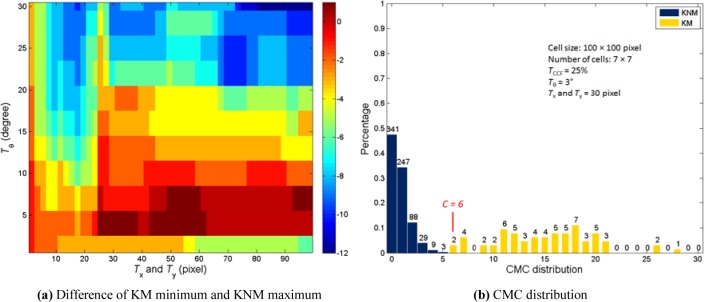
780 correlations result based on *T*_CCF_ = 25 %.

Figure 7a shows the correlation results using the same process on the 40 cartridge cases comprising 63 KM and the 717 KNM, or a total 780 correlations. The location of the maximum CMC difference of the 780 correlations as shown in Fig. 7a is similar to that of the 190 correlations (see Fig. 6a). However, the maximum CMC difference is reduced from 6 (Fig. 6a) to 1 (Fig. 7a). Using the same thresholds as Fig. 6, the CMC distribution of 780 correlations is shown in Fig. 7b. Compared with the results of the training set (see Fig. 6b), the distribution pattern of the KNM set does not have obvious change but the minimum CMC number for KM correlations is 6.

As the results show, the 717 KNM correlations have a similar distribution and same maximum CMC number with the 180 KNM correlations. The CMC method has a good stability of identifications for non-matching samples. This feature has the potential to be of great use to the forensic science of firearm and toolmark identification. The forming of toolmarks on cartridge cases is a complex process. In actual casework practice, features of the matching samples may be widely different. The observation that the CMC distribution of 63 KM correlations were shifting left under the same conditions is expected.

## 5. Discussion and Conclusion

The CMC method and the proposed numerical identification criterion (*C* = 6) for ballistics identification of breech face impressions are validated by 780 correlations of pair-wise optical images of a set of 40 cartridge cases taken under top ring lighting condition. These images are correctly identified by using the CMC method without false identification (false positive) or false exclusion (false negative) identification. The non-matching results showing a low CMC number and a stable CMC distribution help support that the CMC method is feasible for ballistics identification. Further, the results of this experiment show that the CMC method can be applied to both optical images and 3D topography images.

The results can be improved by optimizing the parameters and the correlation strategy. We plan to develop additional improved methods in future work. We also plan to use the CMC method for correlations of firing pin and ejector mark impression signatures.
